# Psychosocial maladjustment arising from workplace sexual behavior directed at adolescent workers

**DOI:** 10.1080/21642850.2019.1653188

**Published:** 2019-08-11

**Authors:** Karen L. Sears, Dennis R. Papini

**Affiliations:** a Department of Psychology, College of Arts and Science, Western Illinois University, Macomb, IL, USA; b Office of Vice Chancellor for Academic Affairs and Provost, University of Illinois at Springfield, Springfield, IL, USA

**Keywords:** Sexual harassment, adolescents, coping, gender role strain, maladjustment

## Abstract

**Objective:** The objective of the current study was to examine psychosocial maladjustment related to adolescents’ appraisals of workplace sexual behavior.

**Method:** High school aged adolescents with formal work experience completed a survey containing a battery of scales.

**Results:** Descriptive statistics addressing frequency of exposure showed that 45% of adolescent men reported at least one incident of sexual behavior directed at them personally, and 24% of adolescent women reported the same. Results further indicated that adolescent men reporting a positive experience after being targeted by direct sexual behavior at work also showed signs of internal maladjustment, such as depression and anxiety.

**Conclusions:** Gender role strain model, which suggests that the male adolescents experienced trauma when conforming to hyper-masculine norms that call for acceptance of sexual behavior, was offered to explain why male adolescents differed from female adolescents in associations between sexual behavior appraisal and maladjustment.

The International Labour Organization (ILO) is a global agency dedicated to setting labor standards and advocating on behalf of decent working conditions for all people. One segment of the organization tracks data specifically targeting labor performed by children under the age of 18. According to this source, 141 million adolescents aged 15–17 worldwide are economically active (International Labour Organization, 2002). Stated another way, 42% of the adolescents aged 15–17 around the world are engaged in some form of paid employment. The vast majority of adolescents concentrate in very few sectors of the labor force; for example, sales, clerical, service, low-skill labor, and farm and industrial jobs (Stern & Nakata, 1989; U.S. Department of Labor, 2000). Adolescent-concentrated jobs, in comparison to the general labor market, tend to be temporary and seasonal, provide fewer fringe benefits, require little independent thought or action, offer less variety of skill use and activity, and provide relatively less social contact and opportunity for learning (Greenberger, Steinberg, & Ruggiero, 1982; Stern & Nakata, 1989). A particularly noteworthy feature of today’s work context for teenagers is diminished intergenerational contact between teens and adults, thereby reducing the opportunities for beneficial mentoring that contact with a caring and accessible adult could afford an employed teen (Greenberger & Steinberg, 1981). Adolescent workers tend to be employed in adolescent-dominated environments, populated by peers and supervisors who are very similar in age (Staff, Messersmith, & Schulenberg, 2009). Consequently, the workplace promises to be a target-rich environment for socializing with other adolescents and young adults (Staff et al., 2009; Staff & Schulenberg, 2010). Along with the rewards of workplace socialization for adolescents, come the associated risks as well (Frone, 1999; Steinberg & Cauffman, 1995). The work environment exposes adolescents to a new, relatively less circumscribed and protected, venue with all of its associated undesirable behaviors, such as sexual harassment.

A major objective of the current research was to examine the *subjective appraisal*, an individual’s judgment of a behavior and its degree of threat to oneself, of adolescent workers and its impact upon psychosocial maladjustment for these adolescents engaged in their first formal jobs. In the current study, we were particularly interested in examining relations among constructs for *adolescent* employees, arguably the most vulnerable population for sexually harassing behaviors due to reduced status associated with younger age (Sears, Intrieri, & Papini, 2011). Much of the documentation for prevalence of sexual harassment has focused on school-based activity. For example, Hill and Kearl’s (2011) comprehensive report, on behalf of the American Association for University Women, on prevalence rates indicated that 48% of the students surveyed (in grades 7–12) reported having experienced some form of sexual harassment at school. More to the point, Fineran’s (2002) descriptive analysis of sexualized behavior *at work* affirmed that adolescent employees are over-represented as targets. In her sample, nearly two-thirds of high-school age women, and one-third of adolescent men of the same age, reported having experienced at least one form of sexualized behavior at work. Sears et al. (2011) contended that the key factor influencing outcomes for adolescent workers targeted by work-based sexually harassing behavior is the person’s *subjective appraisal,* i.e. their judgment about whether the behavior is a threat to their personal well-being (Lazarus & Folkman, 1984).

## Appraisal of workplace sexual behaviors

Berdahl and Aquino (2009) examined the novel proposition that sexual behavior at work can be evaluatively appraised by targets as fun and harmless (Pierce, Byrne, & Aguinis, 1996; Powell & Foley, 1998). In other words, not all sexually-oriented behavior at work is regarded as harassing, or threatening to one’s well-being, in accord with prevailing beliefs. Indeed, non-harassing sexual behavior has been the focus of very little empirical attention (Berdahl & Aquino, 2009). There are a few exceptions. For instance, Berdahl and Aquino (2009) reported that some employees enjoyed sexual behaviors at work, though they did not necessarily gain desirable outcomes from those encounters. In yet another study, Sears et al. (2011) reported that adolescents, in particular, were disinclined to label sexual behavior as ‘harassing.’ When asked to label the sexual behavior experienced at work as ‘harassing,’ very few adolescent workers were likely to use that label. Instead, the authors reported that adolescents occasionally perceive sexual behavior at work as mere playful banter, designed to enhance energy levels and intrinsic interest in otherwise menial job tasks (Sears et al., 2011).

The central construct used by these authors to represent adolescents’ responses to sexually-oriented behavior at work was *appraisal* (Sears et al., 2011). Appraisal is borrowed from classic stress theories, and represents an individual’s process by which that person judges the degree of personal threat to one’s well-being posed by a given social interaction (Lazarus, 1999; Lazarus & Folkman, 1984; Vaile Wright & Fitzgerald, 2007). The initial step in reaction to a social situation is known as the *primary* appraisal, during which an individual makes a virtually automatic emotional assessment of whether a social event, such as sexual behavior at work, threatens well-being. One explanation posed for more positive appraisals is that adolescents are still at a developmental stage where they are learning appropriate sexual interaction norms, thus are less inclined to view such behavior as harassment (Bremer, Moore, & Bildersee, 1992). To gain a more nuanced examination of adolescents’ appraisals, we directed attention to reactions resulting from two forms of workplace sexual behavior: Ambient sexual behavior, and direct sexual behavior.

## Ambient sexual behavior and direct sexual behavior

Both sexual harassment law and research have acknowledged a distinction among types of sexual behavior, herein referred to as *ambient sexual behavior* (ASB) and *direct sexual behavior* (DSB; Berdahl & Aquino, 2009; Equal Employment Opportunity Commission, 1980). ASB is comprised of a range of behaviors; such as sexually oriented jokes, language, and materials; that contribute to a sexually charged work environment. In contrast, DSB refers to a set of behaviors, such as sexualized requests and remarks, which directly target an individual(s) in the workplace.

We draw upon this distinction for purpose of extending the findings of Berdahl and Aquino (2009), who explored *adult* men’s and women’s disparate reactions to ASB and DSB, as a function of a combination of both one’s own sex, and sex of the actor initiating the sexual behavior at work. These scholars explained that the fundamental motive for sexual harassment is the application of power (Berdahl, Magley, & Waldo, 1996; MacKinnon, 1979). For purpose of the current study, we reasoned that all forms of sexual behavior, whether ambient or direct, would reflect a power dynamic and remind adolescent women of their subordinate position in the workplace. Therefore, we expected to find similar patterns of ASB appraisal-maladjustment, and DSB-maladjustment, relationships among adolescent women. Adolescent men, on the other hand, were expected to demonstrate more pronounced DSB-outcome relations, given that DSB amounts to direct sexual comments and advances, which would serve to threaten adolescent men as targets of such attention – moreso than the diffused form of sexual attention of ASB (Berdahl, 2007; Stockdale, Visio, & Batra, 1999).

## Psychosocial maladjustment as a consequence of ASB and DSB appraisal

We posited that an individual’s evaluative appraisal potentially influences that person’s psychosocial outcomes. Indeed, several studies utilizing adult populations have linked sexual harassment and victim’s emotional, psychological and behavioral responses, such as sadness, depression, anxiety, and interpersonal withdrawal (Collinsworth, Fitzgerald, & Drasgow, 2009; Willness, Steel, & Lee, 2007). Similar relationships between exposure to sexual harassment and difficulty in adjusting has been reported among adolescent populations as well (Goldstein, Malanchuk, Davis-Kean, & Eccles, 2007; Hand & Sanchez, 2000). Drawing from the adolescent health literature, the construct of *psychosocial maladjustment* reflects the extent of psychological and behavioral difficulties in response to events of the adolescent years, and it served as the criterion variable in the current study. Adolescents struggling to adjust to life events are conceptualized as suffering from two broad categories of maladjustment (Achenbach, 1991; Felix & McMahon, 2006). *Internal maladjustment* reflects a variety of psychological and somatic symptoms, such as anxiety and depression. *External maladjustment*, in contrast, reflects a set of behavioral and social problems, such as delinquency and aggression. Several empirical sources have related adolescents’ victimization by peers to anxiety and depression (Craig, 1998), school avoidance and loneliness (Kochenderfer & Ladd, 1996), and low self-regard (Egan & Perry, 1998). Felix and McMahon (2006) reported that exposure to sexual harassment was related to both internal and external forms of psychosocial maladjustment among a pre-adolescent sample. Drawing from this research base, we chose to include internal maladjustment, as well as external maladjustment, as the two categories of outcome variables in the current study.

## Internal coping as a moderator of the appraisal – maladjustment relationship

For purpose of this study, we were most interested in the subjective appraisal of adolescent workers in response to a potential stressor, such as sexual behavior at work, and the outcomes realized for that individual. We treated the individual’s self-report of evaluative appraisal, in response to a question about how negative or positive a given social interaction was for the person, as the operational definition of *primary appraisal*. Measures of ASB appraisal and DSB appraisal were assessed separately in order to seek any contrasting relationships to consequences, for male and female adolescent workers.

Yet, following the primary appraisal, the individual undergoes another step, known as the *secondary appraisal*, or the more conscious cognitive determination of response to the perceived stressor (Lazarus, 1999; Lazarus & Folkman, 1984). The secondary appraisal is designed to impose some sort of meaning on a social event, such as a potentially threatening workplace sexual behavior, resulting in rationalized judgments about how to interpret and respond to that behavior. Several forms of judgment may be triggered upon exposure to a sexually-oriented social event at work, but we were particularly interested in examining a person’s degree of internal coping. *Coping*, in general, is comprised of the cognitive and behavioral efforts to manage a social event considered to be demanding upon one’s resources (Lazarus & Folkman, 1984). Workplace sexual behavior, particularly that behavior which directly targets an adolescent, is considered to be a sufficiently novel social event that would stretch the personal resources of the adolescent worker, thus requiring attempts at coping (Magley, 2002).

Here, we draw from past research that examines coping as a mechanism for adapting to unwanted sexual harassment (Fitzgerald, Swan, & Fischer, 1995; Gruber & Fineran, 2007; Knapp, Faley, Ekeberg, & Dubois, 1997; Magley, 2002; Malamut & Offermann, 2001). A distinction is drawn between *external* (problem-focused) coping and *internal* (emotion-focused) coping (Magley, 2002). Whereas external forms of coping are motivated by a desire to directly manage a situation (e.g. avoidance, appeasement), *internal* coping is a strategy designed to influence the emotions and cognitions arising from the social situation. Specific examples of internal coping mechanisms include, but are not limited to, ignoring the social behavior, denial, trivializing the situation, and re-labeling the behavior as something more benign. We were interested in utilizing the concept of internal coping, in particular, as a cognitive form of secondary appraisal in response to work-based sexual behaviors (Magley, 2002). Internal coping was treated in our model as a moderator variable, along with gender, in the association of primary ASB (DSB) appraisal to psychosocial maladjustment for the adolescent worker.

Level of internal coping is predicted to modify, and likely accentuate, the degree to which appraisal and maladjustment are associated with one another. This prediction draws from classic emotion literature which generally finds that when in-depth cognitive processing in search of meaning and interpretation accompanies an emotional state, such as the emotion accompanying a novel social situation like work-based sexual attention, there tends to be a strengthened emotion-outcome relationship (Forgas, 1995, 2002).

## Differing patterns for adolescent men and women

In considering the predicted direction of appraisal-maladjustment relationship among the adolescent men in our sample, we utilized concepts from Pleck’s (1981) *gender role strain model*. Pleck (1981, 1995) argued that male *gender role trauma* results from the early socialization experiences serving to mold adolescent men in society’s image of ideal masculinity. According to the model, even the successful fulfillment of expectations associated with this image, such as pressures in the workplace to conform, leads to undesired side effects for the male adolescent. Empirical examination has reported that adolescent men’s strong adherence to traditional male roles was associated with alcohol consumption, drug use, problems with the police, and reckless interpersonal sexual behaviors (Pleck, Sonenstein, & Ku, 1993a, 1993b). Additional research has revealed relationships between adherence to male roles and anger, anxiety, and depression (Eisler, Skidmore, & Ward, 1988; Good & Mintz, 1990; Sharpe & Heppner, 1991).

Applying this gender role trauma explanation to the current research model, we reasoned that adolescent men overtly expressing positive appraisals of sexualized activity in the workplace, in adherence to hyper-masculine normative expectations to accept such behavior, would realize higher levels of maladjustment as the result of trauma strain. The adolescent male suffers undesirable psychosocial effects due to a socialization process instilling traditional masculine ideology, which is inherently traumatic (Levant, 2011; Levant & Pollack, 1995). In effect, we predict that adolescent men will demonstrate *appraisal-incongruent* psychosocial consequences due to the trauma strain realized when trying to reconcile experienced sexual attention (especially direct sexual behavior – DSB) with the normative pressures to accept that attention. We reasoned that an individual relying on an internal, cognitive means of coping further exacerbates the gender role trauma strain associated with normative expectations that an adolescent male should be able to gracefully manage, and even accept, a sexually-charged environment (Sears et al., 2011; Stockdale et al., 1999). In summary, we predicted for adolescent men a *positive* association between appraisal and psychosocial maladjustment, such that reported positive evaluations will be related to higher levels of maladjustment. Moreover, the appraisal-maladjustment relation for adolescent males is expected to be more pronounced among those men reporting high internal coping, as opposed to men who do not. Given that DSB should be more traumatic an experience than ASB, we expect a differentiated pattern for appraisal of DSB and ASB.

In contrast, adolescent women are not burdened with the same gender role expectations to be accepting of sexual behavior at work (Nelson & Burke, 2002). That is, adolescent women are not expected to experience the same gender role trauma strain as that of adolescent men. Consequently, women’s reactions to sexual behavior at work will be closely aligned with their emotional appraisal, and with their cognitive interpretations (such as internal coping). Indeed, past research has revealed that women’s tendencies to cognitively cope with gender discrimination and harassment are related to decreased well-being over time (Collinsworth et al., 2009; Foster, 2009). Applying these general principles and past research to the current study, adolescent women would be expected to demonstrate a *negative* relationship between appraisal and psychosocial maladjustment; i.e. higher levels of positive appraisal would be related to lower levels of maladjustment. Further, because coping is designed for the express purpose to alleviate distress, this appraisal-maladjustment relation should be more pronounced among adolescent women who engage in internal coping strategies, relative to those adolescent females who do not (Gruber & Fineran, 2007; Leaper, Brown, & Ayres, 2013). In other words, adolescent women are expected to show *appraisal-congruent* psychosocial outcomes. Moreover, a negligible difference between ASB appraisal and DSB appraisal is anticipated because sexual behavior at work, whether ambient or direct, would nonetheless be perceived by adolescent women as evidence of subordination and personal threat (Berdahl & Aquino, 2009).

*Hypothesis 1:* Internal coping and gender serve as moderators in the relationship between ASB appraisal and internal maladjustment. Specifically, adolescent men will demonstrate a positive relation between ASB appraisal and internal maladjustment, and that relation will be pronounced for adolescent men who report internal coping. Conversely, adolescent women will demonstrate a negative relation, and that relation will be pronounced for women who report internal coping.

*Hypothesis 2:* Internal coping and gender serve as moderators in the relationship between ASB appraisal and external maladjustment. Specifically, adolescent men will demonstrate a positive relation between ASB appraisal and external maladjustment, and that relation will be pronounced for men who report internal coping. Conversely, adolescent women will demonstrate a negative relation, and that relation will be pronounced for women who report internal coping.

*Hypothesis 3:* Internal coping and gender serve as moderators in the relationship between DSB appraisal and internal maladjustment. Specifically, adolescent men will demonstrate a positive relation between DSB appraisal and internal maladjustment, and that relation will be pronounced for adolescent men who report internal coping. Conversely, adolescent women will demonstrate a negative relation, and that relation will be pronounced for women who report internal coping.

*Hypothesis 4:* Internal coping and gender serve as moderators in the relationship between DSB appraisal and external maladjustment. Specifically, adolescent men will demonstrate a positive relation between DSB appraisal and external maladjustment, and that relation will be pronounced for men who report internal coping. Conversely, adolescent women will demonstrate a negative relation, and that relation will be pronounced for women who report internal coping.

## Method

### Participants

A total of 498 U.S. American high school aged individuals completed a paper-and-pencil survey. Among the 498 respondents, 282 were women and 213 were men (3 were unreported). The majority of respondents were upperclassmen in high school (261 seniors, 114 juniors, 75 sophomores, 36 freshmen, and 12 unreported), with an average age of *M* = 17.04 (*SD* = 2.77) years. The racial and ethnic distribution among members of the sample was as follows: 372 White/Caucasian, 16 Asian, 20 Hispanic, 71 African American, 7 Native American, 8 ‘other,’ and 4 unreported. The single eligibility requirement for this high school age sample was that the participant had to be employed currently in a formal job. The average number of jobs reported by members of the sample was *M *= 1.57 (*SD *= 1.14).

Among that sample, 264 individuals reported having experienced at least one form of ASB (139 men, 124 women, 1 unreported; average age *M* = 17.12; 72.3% White/Caucasian); and 164 individuals reported at least one form of DSB (96 men, 67 women, 1 unreported; average age *M* = 16.92; 67.9% White/Caucasian). Given the purpose of our study to examine predictors and consequences of ASB and DSB, the analyses reported hereafter were conducted exclusively with the sub-sample of respondents reporting experience with ASB or DSB, as appropriate.

### Measures

An institutionally-approved paper-and-pencil survey booklet was supplied to each research recruit, characterizing the project as an ‘Adolescent Employment Survey.’ The cover page indicated that the purpose of the research was to learn about their experiences in a job currently held. The survey began with a set of questions about the respondent’s demographic information and employment profile (e.g. current age, age at time of first formal job, type of job, number of weekly hours at job). Because potentially sensitive questions were being asked, the cover page also included information to respondents about where they can go for help if impacted by the contents of the survey. Thereafter, a series of scales were administered, in the same order appearing herein.

### Psychosocial maladjustment

Psychosocial adjustment was measured with the Youth Self-Report, a 61-item bi-dimensional instrument (Achenbach, 1991). The Youth Self-Report was designed and validated for use with adolescent individuals displaying emotional and behavioral problems. The instrument was comprised of two scales, *internal maladjustment* measuring degree of anxiety, depression, withdrawal, and somatic complaints (31 items; sample items include ‘I feel lonely,’ ‘I am shy,’ and ‘I feel overtired’); and *external maladjustment* measuring degree of delinquent and aggressive behaviors (30 items; sample items include ‘I lie or cheat’ and ‘I get in many fights’). Each item was measured on a rating scale of 0 = not true, 1 = somewhat or sometimes true, or 2 = very true or often true. The two subscales were summed, respectively, serving as two separate dependent variables. High scores are interpreted as high levels of maladjustment. Coefficient alpha for the internal maladjustment subscale was *α* = 0.90. Coefficient alpha for the external maladjustment subscale was *α* = 0.88.

### Appraisal of sexual behavior

In keeping with the measures used by Berdahl and Aquino (2009) for Ambient Sexual Behavior (ASB) and Direct Sexual Behavior (DSB), we selectively extracted items from the SEQ (Sexual Experiences Questionnaire; Stark, Chernyshenko, Lancaster, Drasgow, & Fitzgerald, 2002) that could be subjectively experienced either negatively or positively. Each item was prefaced by the following prompt: ‘While working at your current place of employment, have you been in a situation where a coworker or supervisor ___.’ The ASB subscale was comprised of three items designed to complete that sentence: ‘ … told sexual stories or jokes?’; ‘ … made attempts to draw you into a discussion of sexual matters (for example, attempted to discuss or comment on your sex life)?’; and ‘ … displayed, used, or distributed sexist or suggestive materials (for example, pictures, stories, or pornography)?’ The DSB subscale was comprised of three items: ‘ … made remarks about your appearance, body, or sexual activities?’; ‘ … touched you in a way that felt sexual in nature?’; and ‘ … made attempts to stroke, fondle, or kiss you?’

For each of these six sexual behaviors, a pair of questions followed. First, the questionnaire assessed how often a participant experienced each of these behaviors on a Likert scale from 0 = ’never’ to 4 = ’most of the time.’ Then, among those who responded that they had experienced the behavior at least once (i.e. provided any response other than 0 = never), we asked an additional question about their appraisal of the behavior. Respondents were asked, ‘Indicate how negative or positive the experience(s) had been for you’ on a 1 (very negative) to 5 (very positive) scale. To form a measure of ASB appraisal that could be compared across participants, regardless of number of items endorsed, we then formed an index as follows: Scores for the three ASB appraisal items were summed, and that sum was divided by the number of items in the ASB subscale that were endorsed, thereby forming a proportion. The resulting index value thus represented respondents’ appraisal of the ASB scale items, with higher scores representing more positive appraisals. The coefficient alpha for ASB appraisal was *α* = 0.70. The same appraisal index was calculated separately among the three DSB items, and its coefficient alpha was *α *= 0.70.

### Internal coping

Finally, we assessed respondents’ degree of internal coping. To accomplish this, we used the internal coping sub-scale of the Coping with Harassment Questionnaire (Magley, 2002). This scale assesses forms of cognitive engagement (such as relabeling, appeasement, and self-blame; 15 items), and cognitive disengagement (such as denial, detachment, and endurance; 15 items). Participants responded on a 5-point Likert scale the extent to which each item was descriptive of their reactions; high scores represented items that were highly descriptive. Scores were summed across the 30 items to obtain a total internal coping score. The coefficient alpha for internal coping was *α* = 0.91.

### Procedure

A select and trained team of undergraduate students was utilized to carry out the tasks of recruiting participants and administering surveys. The recruiters’ tasks were to (a) recruit eligible high school age sibling(s) or acquaintance(s) to complete the survey; and (b) follow a precise protocol in administering surveys, and securing the acquired survey results.

The cover page of the survey disclosed the purpose of the study, provided informed consent information, and indicated an eligibility requirement. To be eligible, the survey clearly indicated that a high school aged participant was required to be employed in some type of formal job. The cover page further disclosed that survey questions would be asking about one’s experiences relevant to a current job, including exposure to sexualized behavior, coping strategies, and personal well-being. Spaces were available on the cover page for signature of the high school respondent, as well as signature of a parent.

Prior to approaching prospective respondents, our research team recruiters were required to attend training sessions to ensure adherence to a precise protocol. Recruiters first approached parents/guardians to gain assurance and consent on three fronts: (a) their adolescent child did indeed meet the eligibility requirements of being in high school and having a formal job; (b) the parent or guardian had examined the contents of the survey and provided explicit consent for their child to participate, as signified by a signature on the cover page; and (c) the parent or guardian was willing to oversee the survey administration and assure that their child was allowed complete privacy in filling out the survey and that the resulting survey was indeed completed by the eligible child.

Recruiters also operated from a standardized recruiting script disclosing participant role and rights, the general purpose of the study, and instructions for completion, when gaining assent by minor child participants as signified by their signature on the cover page. The recruiter script also required that the recruiter discuss with the adolescent child the meaning of voluntary participation for the benefit of others, and to gain assurance that the respondent was comfortable with the survey contents and their role as a participant. Each adolescent respondent was then supplied with a sealable manila envelope, in addition to the questionnaire packet. They were assured confidentiality, and the role of their parent/guardian was also explained. Research team recruiters later retrieved completed surveys, enclosed within sealed envelopes.

### Ethics Statement

The study was approved by the Institutional Review Board of South Dakota State University (Approval # IRB-1207006-EXP).

## Results

Preliminary analysis began with an examination of the means, standard deviations, intercorrelations, and coefficient alpha values among the major variables in this model. The resulting values are available in Table 1. All coefficient alpha values were reasonably high, meeting generally accepted standards. Prior to testing hypotheses, we also inspected our five major variables to verify the assumption of normality. In this analysis, skewness and kurtosis measures indicated that the measures were reasonably normal in shape, and visual inspection of graphs of the variables confirmed that none of the variables deviated significantly from normality. The average ASB appraisal score across only those respondents reporting any degree of exposure to sexual behavior was *M *= 3.06 (*SD* = 0.74), indicating that respondents, on average, were neutral in their evaluations of ASB on a scale of 1 (very negative) to 5 (very positive). The average DSB appraisal score was *M* = 2.98 (*SD* = 0.96), on the same scale, indicating that respondents were neutral in their evaluations of DSB. As displayed in Table 1, male respondents demonstrated generally higher appraisal levels for both types of sexual behavior (*M* = 3.16 for ASB, *M *= 3.16 for DSB); whereas female respondents demonstrated lower appraisal levels (*M* = 2.96 for ASB, *M *= 2.86 for DSB).

**Table 1.** Intercorrelation matrix.

	Men means (SD)	Women means (SD)	1	2	3	4	5
1 ASB appraisal	3.16 (0.59)	2.96 (0.84)	.70	.33**	.03	−.16	−.02
2 DSB appraisal	3.16 (0.79)	2.86 (1.05)	.28	.70	−.14	−.09	.03
3 Internal coping	63.08 (19.83)	58.85 (19.77)	.05	−.03	.91	.26**	.21**
4 Internal maladjustment	8.85 (8.20)	11.94 (8.74)	−.04	.03	.19	.90	.54**
5 External maladjustment	11.17 (8.42)	10.25 (7.37)	.03	.24	.24*	.57**	.88

Note: Coefficient alpha on diagonal. Intercorrelations for men are on lower diagonal; intercorrelations for women are on upper diagonal.

*p < .05; **p < .01.

### Hypotheses 1 and 2: internal coping and gender as moderators of ASB appraisal – maladjustment relationships

Hypotheses 1 and 2 predicted that internal coping and gender would serve as moderators of the relation between ASB appraisal and internal/external maladjustment. A series of hierarchical regressions were conducted for each of the moderators and for each outcome variable, respectively. For example, for the outcome variable internal maladjustment (Hypothesis 1), the simple effect for ASB appraisal was added in the first step, the simple effects for internal coping and for gender were added in the second step, all combinations of two-way interactions were added in the third step, and the ASB appraisal × internal coping × gender three-way interaction was added in the fourth step. Prior to creating the interaction terms, the predictor (ASB appraisal) and internal coping moderator were centered by subtracting each value from its respective mean, for the purposes of reducing multicollinearity and achieving better estimates of the interaction terms (Aiken & West, 1991; Cohen, Cohen, West, & Aiken, 2003). Support for Hypotheses 1 and 2 would come in the form of a significant three-way interaction term. No effects emerged in the final step for either of the two forms of psychosocial maladjustment, thus failing to support Hypotheses 1 and 2.

### Hypotheses 3 and 4: internal coping and gender as moderators of DSB appraisal – maladjustment relationships

Hypotheses 3 and 4 predicted that internal coping and gender would serve as moderators of the relation between DSB appraisal and internal/external maladjustment. The aforementioned set of hierarchical regression analyses was performed again, with the exception of using DSB appraisal as the predictor. We expected three-way interactions among DSB appraisal, internal coping, and gender for each of internal and external maladjustment, in testing Hypotheses 3 and 4, respectively.

**Figure 1.**
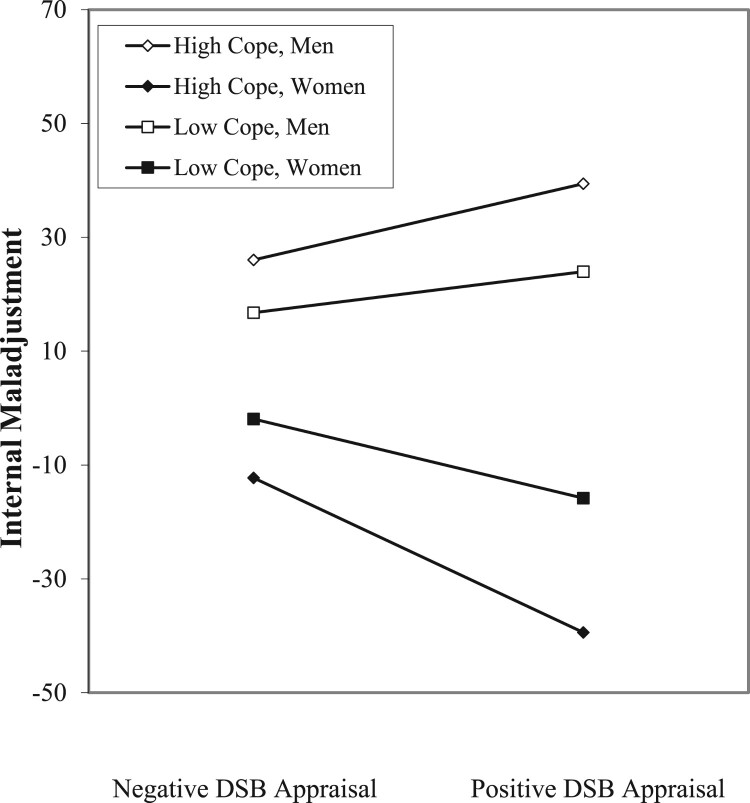
Internal maladjustment as a function of DSB appraisal, internal coping, and gender.

A significant three-way interaction emerged for internal maladjustment in our examination of Hypothesis 3. Details from this analysis may be viewed in Table 2. For interpretation of this interaction, we used a graphing technique examining the simple slope for internal maladjustment regressed on DSB appraisal for low and high levels of internal coping, and for men and women, respectively (Aiken & West, 1991). Figure 1 displays the nature of the DSB appraisal × internal coping × gender interaction on internal maladjustment, indicating a positive association between DSB appraisal and internal maladjustment among adolescent men (a difference-of-slope test indicated no significant difference between the male/high coping and male/low coping groups; Dawson & Richter, 2006). Among women, on the other hand, there was a negative relationship between DSB appraisal and internal maladjustment, and that negative association was more pronounced for adolescent women who engaged in internal coping (according to a difference-of-slope test).

**Table 2.** Summary of hierarchical multiple regression results: DSB appraisal, internal coping, and gender on internal maladjustment (Hypothesis 3) and external maladjustment (Hypothesis 4).

	Internal maladjustment external maladjustment							
	Δ*R*^2^	B	SE(B)	*β*	Δ*R*^2^	B	SE(B)	*β*
Step 1: DSB appraisal	.000	−.17	.94	−.02	.013	.96	.78	.11
Step 2: DSB appraisal	.079	.27	.94	.03	.069	1.15	.77	.14
Internal coping		.14	.05	.27**		.12	.04	.26*
Gender		−3.14	1.90	−.15		−.24	1.57	−.01
Step 3: DSB appraisal	.002	−.19	3.10	−.02	.053	−3.94	2.47	−.47
Internal coping		.19	.15	.37		.06	.11	.14
Gender		−3.07	1.95	−.15		−.55	1.55	−.03
DSB app × coping		−.01	.05	−.01		.01	.04	.04
DSB app × gender		.37	2.17	.05		3.91	1.73	.65*
Coping × gender		−.04	.10	−.11		.04	.08	.13
Step 4: DSB appraisal	.035	−1.34	3.11	−.13	.011	−4.46	2.51	−.53
Internal coping		.15	.14	.30		.05	.12	.11
Gender		−2.56	1.93	−.13		−.33	1.56	−.02
DSB app × coping		−.28	.14	−.61		−.12	.12	−.31
DSB app × gender		.61	2.14	.08		4.01	1.73	.67*
Coping × gender		−.02	.10	−.07		.04	.08	.15
DSB app × coping × gender		.19	.09	.62*		.09	.08	.35
Total *R*^2^	.116				.146			

*p < .05; **p < .01.

**Figure 2.**
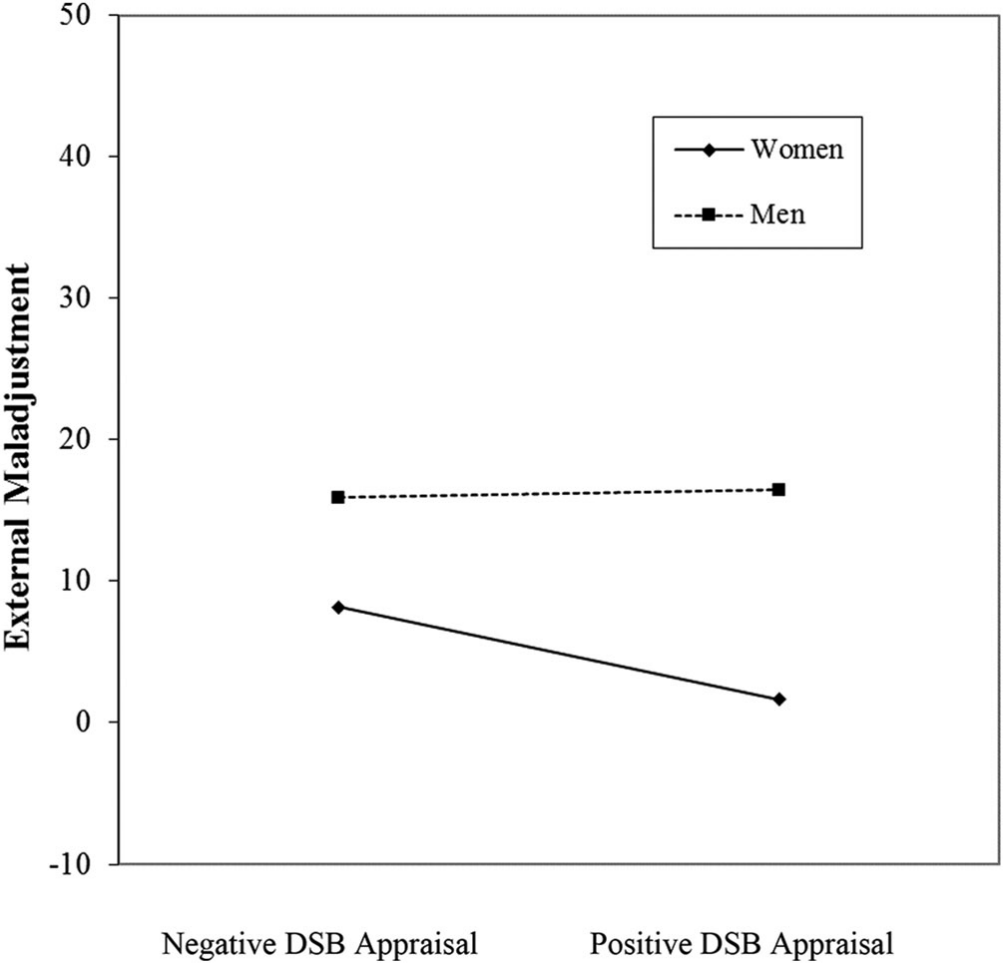
External maladjustment as a function of DSB appraisal and gender.

An analysis also yielded a DSB appraisal × gender two-way interaction in the final step for external maladjustment, thus partially supporting Hypothesis 4 (see Table 2). Adolescent men displayed uniform levels of external maladjustment, regardless of DSB appraisal level; whereas adolescent women showed a negative relationship between DSB appraisal and external maladjustment (Figure 2).

## Discussion

The objective of the current study was to examine psychosocial consequences of adolescents’ appraisals of work-based sexual behavior. This goal was pursued without forcing a negative lens upon sexual behavior at work; rather, evaluative appraisal was measured on a continuum of desirability, allowing the respondent to express whether the behavior was pleasurable or not (Berdahl & Aquino, 2009). Prior research reported that mere exposure to sexual behavior at work, even if it is positively appraised by the target of that behavior, was related to negative consequences for the individual (Berdahl & Aquino, 2009). The current study found a similar trend in that positive appraisal of work-based sexual behavior was not a guarantee for desired psychosocial consequences. We built upon the findings of Berdahl and Aquino (2009) by exploring some of the underlying psychological mechanisms responsible for appraisal-incongruent outcomes. Results of the current research indicated that adolescent men were most likely to show signs of internal maladjustment when positively appraising direct sexual behavior (DSB) at work (i.e. appraisal-incongruent outcomes). Adolescent women, in contrast, demonstrated an appraisal-congruent response to DSB; they showed lowered levels of internal maladjustment with positive appraisal, especially when accompanied by internal coping.

Gender emerged as an important predictor of effects. Although female respondents followed the logical pattern of psychosocial adjustment in relation to positive evaluations of sexual behavior, the same did not hold for men. Male adolescents’ DSB appraisals of workplace sexual behavior were not related to external maladjustment. However, positive DSB appraisal among male adolescents related positively to internal maladjustment, characterized by feelings of anxiety and depression. At first blush, this finding may seem illogical. Why would individuals reportedly enjoying sexual attention also be displaying symptoms of depression? We argue that this finding for adolescent men is supportive of the gender role strain model, wherein male adolescents outwardly endorsing sexual attention are effectively adhering to gender role expectation, yet experiencing a trauma strain resulting from that conformity to hyper-masculine norms. Such norms include the expectation that adolescent men should successfully manage what is regarded as playful sexual banter at work (Mosher & Tomkins, 1988; Sears et al., 2011; Stockdale et al., 1999). This pattern arose for DSB only – and did not extend to ASB – thus suggesting that internal maladjustment experienced by adolescent males experiencing DSB may have resulted from upsetting sexual behavior triggering personal questions about one’s own gender role and heterosexuality, and feelings of powerlessness and self-blame (Platt & Busby, 2009; Street, Gradus, Stafford, & Kelly, 2007).

Beyond the gender role strain model, two alternative explanations assist in gaining insight into mental processes of adolescent men encountering, and positively appraising, sexual behavior at work. One such alternative explanation for reactions by both the target and actor of sexual behavior emphasizes the role of cognitive strategies, such as moral disengagement, to justify positive reactions to what may rationally be considered as undesirable or immoral behavior, such as sexual harassment (Page, Pina, & Giner-Sorolla, 2015). Perhaps males are more susceptible to moral disengagement, relative to females, thus resulting in the discrepancy between reportedly positive appraisals and maladjustment. Yet another alternative speaking to the unusual discrepancy between appraisal and results for the male adolescent employees relies upon the concept of implicit attitudes. Implicit attitudes may better predict effects of workplace sexual attention, compared to the explicit appraisals solicited from members of our sample. On a subconscious level, sexuality has stronger negative cognitive associations than positive ones (Geer & Robertson, 2005). Therefore, employees’ implicit attitudes about sexual behaviors at work may have little association with their overtly expressed opinions. We conclude that adolescent men expressing positive evaluations of DSB may, in fact, harbor negative subconscious attitudes that help to explain the undesirable psychological outcomes (Berdahl & Aquino, 2009).

The adolescent women of our sample, in contrast, were unburdened by the same gender role strains, and therefore realized psychosocial outcomes that were more closely aligned with their feelings about sexualized attention experienced at work. The construct of a *sexual harassment script*, borrowed from similar concepts in the rape literature, aids further in understanding why adolescent females may, rather ironically, be better prepared as workplace newcomers to sexualized behavior in that environment (McMullin & White, 2006; Munson, Miner, & Hulin, 2001; Sears et al., 2011). A sexual harassment script is defined as a pre-established cognition of the events and outcomes associated with a social interaction, such as the exchange of sexually-oriented behavior between two people (Sears et al., 2011). Adolescent women entering the workforce are equipped with a relatively well-defined sexual harassment script that offers predictability and meaning to the social event because they, more than adolescent men, carry a higher probability expectation of being sexually victimized at work (Gagnon & Simon, 1987; Jones & Hostler, 2001; Popovich, Jolton, Mastrangelo, Everton, & Somers, 1995; Sears et al., 2011). The sexual harassment script may protect adolescent women from serious maladjustment effects arising from DSB because it affords the woman a virtually automated means of cognitive processing necessary to capture meaning for the social event (Sakaluk, Todd, Milhausen, Lachowsky, & Undergraduate Research Group in Sexuality, 2014; Wiederman, 2005).

One enlightening aspect of the results was a distinct difference in outcomes arising from ASB, in contrast to DSB. ASB, or ambient sexual behavior, is a more diffused form of sexual behavior that lends to a sexually charged work environment, but is not directed at any one particular individual. Unlike DSB, adolescents’ appraisals of ASB were related to neither internal maladjustment, nor external maladjustment. In hindsight, this non-effect may be instructive. Previous research conducted among adult samples has reported lowered well-being among individuals exposed any form of sexual behavior, even if they reported liking it (Berdahl & Aquino, 2009). We deduce that adolescents arrive at the workplace less well equipped, relative to older adults, with an in-depth understanding and response repertoire when exposed to sexual behavior. Typically, it requires for most people a richer and more varied life experience to gain the depth of knowledge to be better able to interpret upsetting behavior as sexual harassment (Blackstone, Houle, & Uggen, 2014; Bremer et al., 1992). ASB, by its nature, is a more subtle form of sexualized behavior that did not relate to maladjustment. DSB, in contrast, is far more overt, and less open to personal interpretation. Therefore, as we found, one’s threat level in response to DSB is a stronger predictor of psychosocial outcomes, such as depression and anxiety. This finding is supportive of the classic stress models relating appraisal to well-being, at least when DSB serves as that stressor (Lazarus, 1999; Lazarus & Folkman, 1984; Vaile Wright & Fitzgerald, 2007).

Another notable differential in the findings revealed that, although cognitive processing such as internal coping played a role in determining internal maladjustment, no such association was found for external maladjustment. We had made a choice to include only internal coping (not external coping) in our model in adherence to stress models that describe secondary appraisal as a largely cognitive process (Lazarus & Folkman, 1984). Internal coping is comprised of a cluster of discrete mental functions, such as trivializing a social event or relabeling it as something harmless. In hindsight, it makes sense that internal coping mechanisms would more likely be associated with the psychological outcomes of depression and anxiety manifesting from internal maladjustment; and less so with overt behavioral outcomes like delinquency and aggression, more closely aligned with external maladjustment.

Although not addressed in our hypothesis tests, descriptive information derived from our adolescent employee sample may be informative. One issue addressed by the descriptive data is the frequency of exposure that adolescents have to ASB and DSB at work. Adolescent employment may carry some benefits, yet those benefits do not come without a price (Sears et al., 2011). Respondents in the sample revealed unacceptably high levels of exposure to sexual behavior. Specifically, 65% of adolescent males, and 44% of adolescent females, endorsed at least one ASB item. Perhaps more alarming was the incidence rate for DSB: 45% of adolescent men reported at least one incident of DSB, and 24% of adolescent women reported the same. These values suggest that one-quarter of the women, and nearly one-half of the men in our sample, reported that, on at least one occasion, they were verbally or physically accosted at work. Yet these high exposure rates are routinely found in the sexual harassment literature, and we contend that adolescent men and women are especially vulnerable as a result of the relatively low status accompanying youth (Berdahl, 2007; Sears et al., 2011).

Interestingly, the exposure frequency numbers for both ASB and DSB were higher for adolescent men, relative to adolescent women. Throughout most of the literature base on sexual harassment, *adult* women tend to report higher exposure levels than do men (Fitzgerald, Hulin, & Drasgow, 1994). The deviating pattern among adolescents in the current study may be explained in part by the status and normative expectations associated with the male role. Social norms spill over into the workplace, placing pressure upon adolescent men, particularly as they are socialized as newcomers to a work environment, to hold positive attitudes toward sexuality (Kroger, 2000; Stockdale et al., 1999). These interpersonal dynamics would likely manifest in male-initiated, and reciprocated, sexualized banter and behavior.

Additional descriptive data replicated findings in past research. Perpetrators of sexual behavior were more often male, and more often coworkers (Fineran, 2002; O’Leary-Kelly, Bowes-Sperry, Bates, & Lean, 2009). Not surprisingly, DSB from male perpetrators was not welcomed by either adolescent men or women, as it would threaten heterosexual masculinity norms for male targets, and reinforce female subordination norms for adolescent women (Berdahl, 2007; Sears et al., 2011). Finally, our adolescent sample labeled their experience as ‘sexual harassment’ very infrequently. As Blackstone et al. (2014) suggested, developmental maturity is required for a more refined shaping of one’s perceptions of sexualized workplace interactions.

### Limitations and practical implications

All forms of survey research suffer from inherent limitations, and the current investigation is no different. First, generalization is influenced by the limits in the sample drawn. Any interpretations drawn must be qualified by the fact that we gathered responses from inexperienced adolescent workers who tend to concentrate in low-skill and low-pay sectors of the U.S. American labor force, for instance in the service and agricultural industries. Moreover, because instructions restricted respondents to revealing sexual behaviors emanating only from coworkers and supervisors, the results fail to speak to many forms of harassment emitted by customers, vendors, others encountered in the workplace, as well as harassment exhibited in the classroom or other environments in which adolescents congregate (Duffy, Wareham, & Walsh, 2004; Gettman & Gelfand, 2007). The degree of generalizability of results is further limited by our use of opt-in parental consent given that not all parents are necessarily going to agree to research participation, thus resulting in a sample that does not fully represent the population of interest.

Moreover, the cross-sectional nature of the data makes it nearly impossible to draw causal inferences from the findings. Although our theoretical model treated appraisal as a predictor and maladjustment as the criterion, it is quite possible that psychological maladjustment may influence individuals’ views of sexual behaviors in the work environment. The choice to measure predictor and criterion simultaneously results in our inability to rule out this possibility. Finally, the results of the current investigation are limited by a potential for common method variance. The collection of data from one source (adolescent workers) on one occasion, relying only upon self-report measures, increases the risk that findings were influenced by common method variance. To reduce these concerns, we utilized tactics recommended to reduce its likelihood, such as guaranteeing confidentiality; nonetheless, we cannot fully rule out this possibility (Podsakoff, MacKenzie, Lee, & Podsakoff, 2003).

As mentioned previously, our descriptive data indicates that the majority of sexual behavior perpetrators were male, and that DSB deriving from male perpetrators was evaluated very negatively by adolescent males in our sample. Yet, in the course of collecting data, we failed to inquire of respondents the sex of perpetrator for each specific reported behavior. Lacking such fine-grained data made it impossible to make comparisons of respondents’ reactions to cross-sex versus within-sex sexual behaviors. Such analysis may have offered more rich detail about outcomes for adolescent men experiencing unwanted attention from same-sex coworkers. Such an approach may be a welcome addition to research of the future.

Social norms have followed court rulings in stigmatizing sexually-oriented workplace behavior. Yet, it persists, and – at least in some sectors – may be considered something desirable to maintain workers’ spirits. Accumulating evidence indicates that, even when positively appraised, individuals may be profoundly harmed by a sexually charged work environment (Berdahl & Aquino, 2009). Adolescent newcomers are among the most vulnerable to the risks posed by sexual harassment. At a societal level, families, educators, and other sources of socialization are responsible for promoting the healthy and positive aspects of gender roles among developing adolescents as a means of preventing, and mitigating the effects of, such harm. Further, behavioral health care professionals should be sensitized to the work setting’s growing sphere of influence upon youth in their efforts to assess and treat maladjusted behaviors and cognitions.

Results of the current study strongly suggest a need for expanded preventive policies and interventions in workplace environments, keeping the protection of adolescent workers at the forefront. Given the direction and intensity of trends favoring adolescent-age employment, the workplace provides an increasingly important influence upon the emotional, attitudinal, and social development among young workers. One’s initial job experiences may set the stage for adulthood professional socialization and, as such, call for a more conscious, strategic approach to introducing adolescents to work settings. Such an approach may include initiatives like specialized selection and training processes for supervisors in those sectors where adolescent workers tend to concentrate; sexual awareness training and educational programs that emphasize content tailored to the needs of adolescent workers, such as healthy professional relationships; and disciplinary policies designed to send a message that harassment targeting the most vulnerable of workers, such as adolescents, will be met with swift corrective action. Results of the current study have signaled that power differentials, based upon age and gender, continue to be exploited by those in superordinate positions in expressing work-based sexual behaviors. Aside from the strategies already identified, practical solutions might include the establishment of a mentoring system wherein well-trained individuals acquire the explicit role of mentor to adolescent workers. That mentor role may include, in addition to introducing the newcomer to work-related tasks, serving as a guide, protector, and responsible for sharing institutional norms of professional behavior. Results of this study further suggest that respected role models would be pivotal for such a mentoring system to be successful, especially for adolescent entering the work scene. Continued scientific research on the subject matter is called for in order to inform public dialogue and organizational policies designed to protect against the victimization of youth in work settings.
